# Editorial: Musculoskeletal pain phenotypes and personalised pain medicine

**DOI:** 10.3389/fpain.2024.1481839

**Published:** 2024-09-06

**Authors:** Maja R. Radojčić, Lingxiao Chen

**Affiliations:** ^1^ Division of Psychology and Mental Health, Faculty of Biology, Medicine and Health, University of Manchester, Manchester, United Kingdom; ^2^ Department of Orthopaedics, Qilu Hospital of Shandong University, Shandong University Centre for Orthopaedics, Advanced Medical Research Institute, Cheeloo College of Medicine, Shandong University, Shandong, China; ^3^ Department of Biostatistics, School of Public Health, Cheeloo College of Medicine, Shandong University, Shandong, China

**Keywords:** musculoskeletal pain, personalised medicine, phenotype, endotype, theratype, sex hormone deficiency, myofascial unit, antidepressants

**Editorial on the Research Topic**
Musculoskeletal pain phenotypes and personalised pain medicine

In physiology, a phenotype refers to observable characteristics of an individual, resulting from the interaction between the individual's genetic and environmental factors. The concept's extension to pathophysiology makes a foundation for modern personalised medicine, which aims to maximise the benefit and minimise the harm by tailoring treatment strategies. In pathophysiology, a phenotype refers to clinically observable features of a disease, which in healthcare settings means observing morphological and behavioural characteristics. Assessing biochemical and molecular properties implies describing mechanistic and pathophysiological pathways, i.e., defining molecular clusters or endotypes. Endotypes drive the variability of phenotypes and the response to treatment (theratype) (1, 2). Musculoskeletal diseases are heterogeneous conditions with pain as the most common symptom and main contributor to reduced quality of life. Pain is the observable characteristic that varies in type, intensity, frequency, duration, and spread, and has been subjected to different phenotyping efforts (3, 4). In the Research Topic, *“Musculoskeletal pain phenotypes and personalised pain medicine”*, four articles investigate and discuss distinct approaches to accelerate personalised pain medicine.

Sofat and Lambarth reviewed pain stratification options across musculoskeletal diseases. They discussed pain-type phenotypes, i.e., observable nociceptive, nociplastic and neuropathic pain features in inflammatory arthritis, osteoarthritis, back pain and fibromyalgia. They discussed pain-type phenotypes with a reflection on potential mechanisms for endotypes and the recommended treatments in the current UK (National Institute for Health and Care Excellence) and European (European Alliance of Associations for Rheumatology) guidelines for managing these conditions. The review also briefly summarises the genetic basis in musculoskeletal diseases that may play a role in a specific type or overall musculoskeletal pain with directions for further research. Importantly, the role of pharmacogenomics, still rarely considered in the pain field, in tailoring pain treatment options has been illustrated.

Sex differences in musculoskeletal pain have been observed. While any musculoskeletal pain type is more prevalent in women, men tend to report more persistent pain (5). Musculoskeletal pain prevalence is higher in adults older than 50 years after which, it loses the specific pattern. However, hand pain peaks in women aged 50–65 years (5), and most women experience menopause around the age of 50. Gulati et al. in an observational study, examined women referred to a specialist osteoarthritis clinic aiming to establish the link between their age at the final menstrual period and the onset of hand pain. They found that 59% of post-menopausal women developed hand pain within the peri-menopausal period of eight years, defined as starting four years before and ending four years after the final menstrual period. They suggest that this could be a hand pain phenotype potentially related to the female sex hormone deficiency endotype with the potential to personalise the diagnosis and treatment of hand osteoarthritis. They also investigated whether systemic oestrogen-containing hormone replacement therapy (HRT) affected the age of hand pain onset. They found that women who reported using HRT for a minimum of six months but discontinued it more than a year before coming to the osteoarthritis clinic were older when hand pain onset than women who never used HRT. Although the literature on HRT use and musculoskeletal pain is conflicting, as indicated here, starting, taking and stopping HRT at different stages of oestrogen deficiency can play a significant role and should be further investigated.

Non-specific back pain is another heterogeneous high-burden difficult-to-treat musculoskeletal condition. A significant body of research on back pain has focused on the intervertebral disc and facet joint pathologies and like in osteoarthritis of other joints failed to provide reproducible associations between structure and pain (6). Sikdar et al. propose a hypothesis where personalised back pain diagnosis is centred on the myofascial unit. They illustrated the overlap of observable characteristics between non-specific back pain and myofascial pain syndrome and indicated a potential back pain phenotype driven by dysfunction of the myofascial unit. This potential phenotype could correspond to manual therapy and acupuncture theratype. Their proposed diagnostic model of back pain has three sections: assessments of pain characteristics including soft tissue, visceral and somatic pathologies and central sensitisation; evaluation of local and global body movement; and assessment of psychosocial factors including stressors and protective factors. Their theoretical framework seeks further testing through the feasibility of the diagnostic model in clinical settings, replicability of the phenotype, corresponding molecular mechanisms and endotypes, and finally treatment response and improvements in patients’ quality of life.

Patients with musculoskeletal pain usually have comorbidities that contribute to more severe clinical manifestations, impaired quality of life and treatment responses. Depression has been a comorbidity of particular interest due to its prevalence (7), possible bi-directional relationship with pain, and antidepressant use for pain management. Liu et al. discuss important but often neglected considerations for improving personalised pain medicine and optimising antidepressant use (antidepressant theratype). These can be grouped as depression-, pain- and antidepressant-related considerations. While patients with comorbid pain and depression are the most likely to benefit from antidepressants, considering not only the overall severity but specific depression symptoms like low mood or anhedonia can help in selecting the antidepressant, that showed superiority for those symptoms. Pain can manifest in patterns with flares at certain times of the day or over days, which are important parameters for choosing the best medication, pharmaceutical form and dosage regimen. Finally, antidepressants have specific pharmacokinetics and pharmacodynamics, meaning that the time needed for improvements to be observed is different than for other analgesics, and it should be considered to make correct inferences about their effects.

Lastly, the rapid development of artificial intelligence is likely to revolutionise personalised pain medicine soon. Its potential to analyse big data to identify phenotypes and corresponding endotypes and theratypes, accelerate the discovery of new or repurposing of existing medications, optimise dosing and combination therapies, and predict progression are some of the ways to move the field forward (8). However, artificial intelligence lacks independent learning and adaptability. As highlighted in this collection of articles, careful consideration and guidance on numerous minor and major aspects must be provided to artificial intelligence to ensure it delivers the desired outcomes.
